# Stress Detection Using Low Cost Heart Rate Sensors

**DOI:** 10.1155/2016/5136705

**Published:** 2016-06-13

**Authors:** Mario Salai, István Vassányi, István Kósa

**Affiliations:** Medical Informatics R&D Centre, University of Pannonia, Egyetem Utca 10, Veszprém 8200, Hungary

## Abstract

The automated detection of stress is a central problem for ambient assisted living solutions. The paper presents the concepts and results of two studies targeted at stress detection with a low cost heart rate sensor, a chest belt. In the device validation study (*n* = 5), we compared heart rate data and other features from the belt to those measured by a gold standard device to assess the reliability of the sensor. With simple synchronization and data cleaning algorithm, we were able to select highly (>97%) correlated, low average error (2.2%) data segments of considerable length from the chest data for further processing. The protocol for the clinical study (*n* = 46) included a relax phase followed by a phase with provoked mental stress, 10 minutes each. We developed a simple method for the detection of the stress using only three time-domain features of the heart rate signal. The method produced accuracy of 74.6%, sensitivity of 75.0%, and specificity of 74.2%, which is impressive compared to the performance of two state-of-the-art methods run on the same data. Since the proposed method uses only time-domain features, it can be efficiently implemented on mobile devices.

## 1. Introduction

Stress is commonly defined as a feeling of strain and pressure [1]. There is evidence that stress is linked with many diseases, playing a crucial role in the development of cardiovascular diseases [2], diabetes [3], or asthma [4], and it also significantly influences the later course of these diseases. Stress is related to life style; therefore, especially for mobile automated lifestyle counseling and analysis services, the need arises to identify stress automatically during daytime, using physiological data from various sensors. If stress could be reliably and automatically identified, this could directly help users manage stress situations, and it could also be used in medical intelligence applications, for example, in refining blood glucose predictions for diabetics during daytime under influence of stress. However, the available methods for automated stress detection based on low price, ubiquitous sensors, are yet immature. Telemonitoring and self-management systems [5–9] extend the horizons of traditional health care using only point of care measurement data, but the proper interpretation and reliability of the results depend on the reliability of the measured data and the sensor itself.

The two crucial questions related to this problem are as follows:

(i) Whether low price physiological sensors are reliable enough compared to “gold standard” devices accepted by and used in clinical practice.
(ii) Which sensors and algorithms can provide a reliable method for stress detection, at an affordable price and minimal user interaction.

This paper describes our efforts and results in answering these questions. The rest of this paper is organized as follows.  Section 1 gives an overview on applicable technologies and related research. In Section 2, we describe our methods for a small scale device validation and the methods used in the main clinical study that compared heart rate variability (HRV) features between relaxation and mental stress periods. We also present here a simple stress detection algorithm.  Section 3 presents the measurement results of the two studies, with the latter compared with two state-of-the-art algorithms. We conclude the strengths and weaknesses of this study and the newly designed stress detection algorithm in Section 4.

The most commonly used physiological markers of stress are as follows:

(i) Galvanic skin response (GSR): using changes in skin conductivity. During stress, resistance of skin drops due to increased secretion in sweating glands [10].
(ii) Electromyogram (EMG): measuring electrical activity of the muscles. Stress causes differences in the contraction of the muscles which can be used to identify stress [11, 12].
(iii) Skin temperature: changes in temperature of the skin are related to the stress level [13].
(iv) Electrical activity of the heart: the most commonly used stress marker parameters derived from the electrocardiograph (ECG) are the heart rate (HR) and the heart rate variability (HRV) [14].

Stress can also be detected using other, less common markers like accelerometer [15], key stroke dynamics [16], or blinking [17]. It is also common to use a combination of several markers at the expense of an increased system cost and user involvement. Fernandes et al. used GSR and blood pressure (BP) markers [18] for determining stress. Sun et al. describe mental stress detection using combined data from ECG, GSR, and accelerometer [19]. De Santos Sierra et al. in [20] used GSR and HR. Rigas et al. used ECG, GSR, and respiration for detecting stress while driving [21]. Wijsman et al. used ECG, respiration, GSR, and EMG of trapezius muscles for mental stress detection [22]. Riera et al. combined EEG and EMG markers [23]. Singh and Queyam used GSR, EMG, respiration, and HR [24] for detecting stress during driving. Pupil diameter, ECG, and photoplethysmogram were used as markers by Mokhayeri et al [25]. Baltaci and Gokcay used pupil diameter and temperature features in stress detection [26], while Choi used HRV, respiration, GSR, EMG, acceleration, and geographical location [27].

New noncontact methods have also been developed recently to measure stress states. Some of them are hyperspectral imaging technique [28], human voice [29, 30], pupil diameter [31], visible spectrum camera [32], or using stereo thermal and visible sensors [33].

However, observing several markers for identifying stress requires an increasing number of input sensors which in turn increases the overall price and lowers applicability. Prices for heart rate meters range from $70 to $500 USD; GSR devices range from $100 to $500 USD, while EMG devices have price ranges from $450 USD up to $1750 USD. Systems combining multiple sensors are priced much higher. For such systems prices fall between $550 USD and $5700 USD, which already can be considered excessive for a mass telemedical lifestyle counseling application. Therefore, in an ambient assisted living (AAL) system, the number of input sensors should be kept minimal. In the rest of the paper, we focus on the simplest and most researched sensor input, that is, the electrical activity of the heart.

As for the reliability of HRV sensors, there are still surprisingly few reviews reported in the literature to date on the validation of the information content of low cost sensors compared to a clinically accepted “gold standard” device. Some devices that were tested for validity are the SenseWear HR Armband [34], the Smart Health Watch [35], the Actiheart [36, 37], the Equivital LifeMonitor [38], and the PulseOn [39]; and also the Bioharness multivariable monitoring device from Zephyr has been tested for validity [40, 41] and reliability [41, 42]. In all cases, a gold standard device was used simultaneously with the device under test as a method for validating data. However, the validated devices above are high-end devices with a considerable price which present an obstacle for the penetration of telemedicine. For example, the Bioharness device has a price around $550 USD, whereas the price of low cost heart rate meters varies from $70 USD to $100 USD. The lack of reliability tests of low cost devices was our motivation for our device validation study.

For automated stress detection, several methods have been published which use only HRV. In 2008, Kim et al. collected HRV data from sixty-eight subjects [43]. HRV data were collected during three different time periods. High stress decreased HRV features. A maximum classification accuracy of 66.1% was achieved. Melillo et al. in 2011 used nonlinear features of HRV for real-life stress detection [44]. HRV data were collected two times, during university examination and after holidays, on 42 students. Most of HRV features significantly decreased during stress period. Stress detection with classification accuracy of 90% was reported using two* Poincaré* plot features and Approximate Entropy. One year later, using the same data, they designed a classification tree for automatic stress detection based on LF and pNN50 HRV features with sensitivity of 83.33% [45]. In 2013, Karthikeyan et al. created stress detection classifiers from ECG signal and HRV features [46]. Vanitha and Suresh used a hierarchical classifier to classify stress into four levels with a classification efficiency of 92% [47] in 2014. In 2015 Munla et al. used an SVM-RBF classifier to predict driver stress with an accuracy of 83% [48].

## 2. Methods

The main goal of this study is the development of a reliable, robust, low price stress detection method suitable for mobile health applications. The study included two distinct phases. In the first phase (device validation study) we tested the reliability of a low cost telemedical heart rate sensor against an accepted medical device. In the second phase we performed and evaluated a clinical study, using the validated telemedical sensor.

### 2.1. Device Validation Study

#### 2.1.1. Sensor Selection and Measurement Protocol

**Figure 1:**
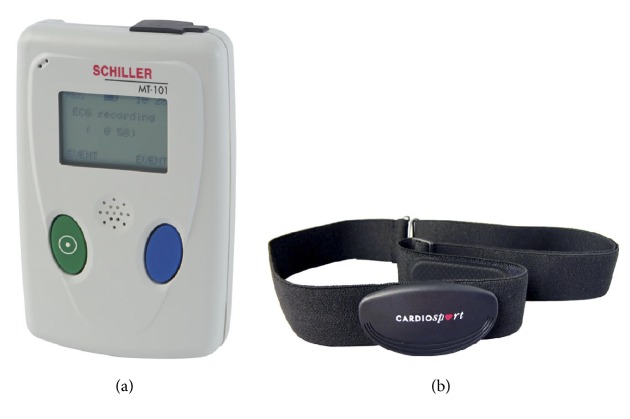
The Schiller MT-101/MT-200 device (a) and the CardioSport TP3 Heart Rate Transmitter device (b).

Among many low cost devices, we have chosen and analyzed CardioSport TP3 Heart Rate Transmitter device, a simple commercial chest belt, as a source of heart rate data, because this is one of the few devices that can measure both heart rate and millisecond accurate RR time interval data. Since this device does not have its own memory for storing data, we used a Nexus 7 tablet with Android version 4.4.2 to connect to the device with the bluetooth 4.0 protocol and store the measured data on the tablet. The reference “gold standard” device was a Schiller MT-101/MT-200 Holter device which was designed for clinical use (see Figure 1).

Five healthy male volunteers used the two devices simultaneously during a 24-hour long period in order to make the measurements (see Figure 1). For chest belt sensors, the temporary detachment or dislocation of the sensor during physical activity or sleep is a common source of errors according to our experiences. Though this problem could be mitigated by using tapes for fixing the device firmly to the body, we felt that such discomfort would not be tolerated in a real AAL situation, so we did not use tapes and used only the daytime 12 hours of the overall signal for analysis. After the monitoring period, we collected the devices and stored the measured data in a unified database.

The protocol was reviewed and approved by the institutional ethics review board in January 2014. The volunteers expressed their informed consent to participate and expressed that they understand the goals of the study before the experiment.

The comparison of the measured data was a hard task due to the different designs of the gold standard and the telemedical device. However, we wanted to compare signals directly in the time domain and also to develop a data cleaning algorithm for the removal of the noisy parts of the CardioSport device measurements, without using the gold standard data. As the chest belt is not firmly attached to the body, even a slight movement of the device could sometimes cause signal loss (especially during sleep). Therefore, we created a software module for synchronization and data cleaning before any further analysis. Data cleaning meant to remove obviously bad data (artifacts) and to keep only “good” data segments of sufficient length, because, as a rule of thumb, both HRV and* Poincaré* plot computation require data chunks of at least 5 minutes. Even though the data cleaning algorithm removes lots of data from the original signal, such a procedure poses no great obstacles for further calculations since we still have enough “good” data during daytime.

#### 2.1.2. Synchronization Procedure

Since the time stamps of the measured records can shift due to device buffering, we used a simple procedure to synchronize the data measured by the CardioSport device with those measured by the gold standard device in order to facilitate their comparison. The algorithm uses a sliding window that passes from the beginning of the chest belt signal to the end and calculates the absolute error between the two signals. When sliding is over, the location of the sliding window with the minimum absolute error is considered as the point where the two signals should be synchronized. This applies only if the correlation of the data in the sliding window and the same amount of data from the gold standard are higher than a minimum set by the user. If these conditions are met, the algorithm copies data from the sliding window into a newly generated third signal which represents the chest belt signal fully synchronized with the gold standard signal. If conditions are not met, the third signal is filled with zeros. At the end, the algorithm extracts all the highly correlated segments from the third signal skipping zero values. Also, a file with all the merged segments is generated for general analysis. The algorithm uses the following 5 main parameters, with their values determined empirically in parentheses:

(i) Window size: how much data is copied from the signal into the sliding window (default: 200).
(ii) Window shift step: number of samples by which we shift the sliding window in each iteration (default: 50).
(iii) Absolute error window: how much data will be used to calculate the minimum absolute error (default: 200).
(iv) Maximum error distance: number of samples by which we shift the absolute error window in order to find the minimum absolute error (default: 1000).
(v) Minimum correlation: minimum correlation, expressed as a percentage, required for the two signals to consider data in the chest belt signal accurate (default: 97%).

#### 2.1.3. Statistical Analysis and Data Processing

We performed time- and frequency-domain analysis and computed the correlation and mean absolute percentage error of the two measurements. We also compared the slope of the scatter plot diagrams of the two measurements.

The time- and frequency-domain analysis for HRV was performed in Kubios HRV analysis software, while the rest of the analysis was performed in Microsoft Excel.

We developed a simple* data cleaning algorithm* to be used in a real telemedical scenario, for automatically finding good parts of the signal, even without gold standard data. This means finding gaps and abnormal values and skipping them. First, we compare the timestamp of each data item with the timestamp of the previous one. If the difference between the timestamps is bigger than 3 seconds, we mark this data as a gap. Three seconds is used for gap detection because the chest belt has a buffering system that can tolerate short detachments of the device from the body. If more than 3 seconds is used, some data could be missing which could cause errors in further data analysis. In the second step we identify abnormal values in the signal. It is important to emphasize that we do not modify the data in any way as this could potentially result in false results in the subsequent analysis. Instead, abnormal values are treated the same way as gaps. The abnormal values are identified by observing the mean value of 20 contiguous samples (10 previous and 10 following ones). If this mean value differs from the value of the current sample by more than 300, we consider it invalid and mark as gap/error in the signal. Finally, we extract the good segments from the signal with a length of more than 5 minutes.

### 2.2. Clinical Study

#### 2.2.1. Measurement Protocol

46 healthy volunteers, mostly university and high school students (27 men and 19 women; average age: 24.6 years), participated in the experiment. The experiment was divided into two parts with a duration of 10 minutes each, so the whole procedure lasted for 20 minutes. In the first part, the participants were asked to try to relax in upright sitting position while listening to relaxation music. The second part of the experiment was a mental task designed to serve as a source of mental stress. We used the Stroop color test smartphone game [49] which is commonly applied to induce mental stress in similar studies. In this game, the user must connect colors to labels at an ever increasing pace. Since controlled breathing and posture have been reported before influencing HRV features, we asked the participants not to control their breathing and to sit still in the same position during the whole experiment. This was also necessary to prevent the detachment of the chest belt from the body. RR intervals were recorded using the CardioSport TP3 Heart Rate Transmitter. The participant was asked about her/his subjective stress levels on a relative scale three times, that is, before the experiment, after the relaxation part, and after the game playing part. The answers along with her/his age and gender were recorded in a simple questionnaire (see Appendices A-B). The reason for such questions was that, though less expected, game playing may be more relaxing for some people than music, and only if we actually succeeded in raising the stress level in the second part compared to the first part, can we expect any algorithm or method to detect the stress. After the recording, the device was unmounted from the participant.

The protocol was reviewed and approved by the institutional ethics review board in January 2014. The volunteers expressed their informed consent to participate and expressed that they understand the goals of the study before the experiment.

After the experiment all data was stored in a unified database, and the data cleaning algorithm described in Section 2.1 was run on both 10-minute parts of each record. Those participants whose records contained no “clean” segments of at least 5 minutes in both parts were excluded from the further analysis. Similarly, we excluded those who—despite our efforts for provoking stress in the experiment—reported no increase of stress level due to game playing.

#### 2.2.2. Statistical Analysis

We used the Kubios software package for getting HRV features and later we analyzed and compared data using the MedCalc software and Microsoft Excel. Wilcoxon paired-samples test was used as a tool for determining significant changes between the two parts of the experiment for the measured values of the HRV features and a *p* value of < 0.05 was considered as significant. Correlation, percentage differences, average percentage differences, and minimum percentage differences were also calculated for all the observed HRV features.

#### 2.2.3. Stress Detection Algorithm

We developed a simple algorithm to detect stress that uses only time-domain HRV features. The reason for excluding frequency-domain features is that they require much more computing power to calculate than time-domain features, an argument that we should consider in a solution designed for mobile devices. We used a combination of the mean HR, pNN50, and RMSSD features to identify stress. A sliding window over the HR signal was divided into four equal parts. We tested various lengths of the sliding window and the shortest width of the window that achieved good result was 560 RR intervals with shift of 20 RR intervals in each step. We used brute force technique to find best threshold values for each HRV feature. As a result, stress is detected by the algorithm if the mean heart rate in the fourth part compared to the first part increases by more than 5%, and RMSSD and pNN50 values decrease by more than 9% in the fourth part compared to the third part. We must emphasize here that this algorithm does not detect rest state. So for the sake of calculating accuracy, specificity, and sensitivity, rest state is considered if stress was not detected. The state of the subject after stressor is also not recognized. Therefore, instead of detecting the subject's physiological state of stress, the purpose of our algorithm is to detect those stressful events which have negative impact on the subject's current state but which may or may not lead the subject into a stressful state. A series of stressful events instead of a single major event can also gradually put the subject into a stressful condition. In a binary classification model this could lead to the false conclusion that only the last event was the one which caused stress, while all the previous events are not taken into consideration and remain hidden.

We note that though it is true that the HRV features may be due to other factors such as depression and mood, we postulate that these factors do not change during the experiment. In contrast, the proposed method uses a well-defined* change* in the HRV features to detect the beginning of the (induced) stressful state; therefore we expect no false positive stress detections due to such factors.

**Figure 2:**
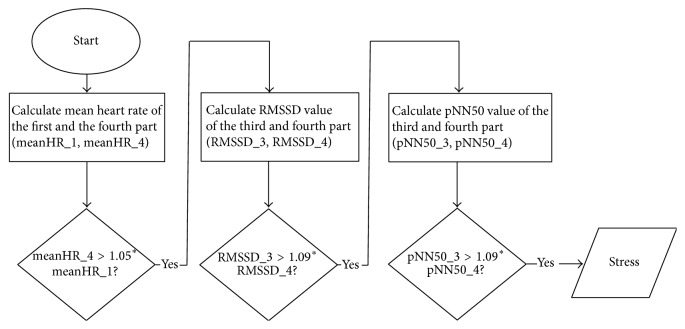
Flow chart of stress detection algorithm.

Figure 2 shows a summary flow chart of the proposed stress detection algorithm which implements the above procedure.

In order to test the power of this algorithm, we compared its performance to two state-of-the-art algorithms from the same author, Melillo et al. [31, 32], on the same dataset.

#### 2.2.4. Performance Comparison to a Linear HRV Algorithm

The algorithm described in [45] uses the pNN50 feature from the time domain and the LF feature from the frequency domain to create a simple classification tree. Stress is detected if LF < 899.58 and pNN50 > 0.9873 or if LF > 277.28 and pNN50 < 0.9873. Restful state is detected if LF > 899.59 or if LF > 277.28 and pNN50 < 0.9783.

For extracting the LF and pNN50 features, we used the same software as the authors of this algorithm, Kubios. Our experiment consisted of two 10 minutes long periods so we extracted two 5 minutes long segments from relaxation part and two 5-minute segments from game playing part. If stress was detected in any one of them, we marked the whole 10-minute part as STRESS. If both parts were detected as REST, then whole 10-minute part was marked as REST.

#### 2.2.5. Performance Comparison to a Nonlinear HRV Algorithm

A stress detection method based on nonlinear analysis [44] was the next algorithm we used. This algorithm uses three nonlinear features: Poincaré plot SD1, Poincaré plot SD2, and Approximate Entropy (En). According to the method, stress is found if

(1) 10.64+203.99·SD1−108.74·SD2−8.26·En0.2>0.

To reconstruct this algorithm we used Microsoft Visual C# to calculate Approximate Entropy based on formula described by authors. A sliding window was used to scan the whole relaxation part as well as the game playing part. If stress was found in any step, we marked the whole 10-minute period as STRESS and, similarly, if rest was detected on all steps of whole part, we marked that part as REST.

In order to compare the performances of the three methods, we computed the accuracy, specificity, and sensitivity for each of them. For this, we registered a true positive result if the method marked the game playing part as STRESS, a true negative result if the relax music part was marked as REST, a false positive result if the relax music part was marked as STRESS, and a false negative result if the game playing part was marked as REST.

## 3. Results

### 3.1. Device Validation Study: Comparison with the Gold Standard

**Figure 3:**
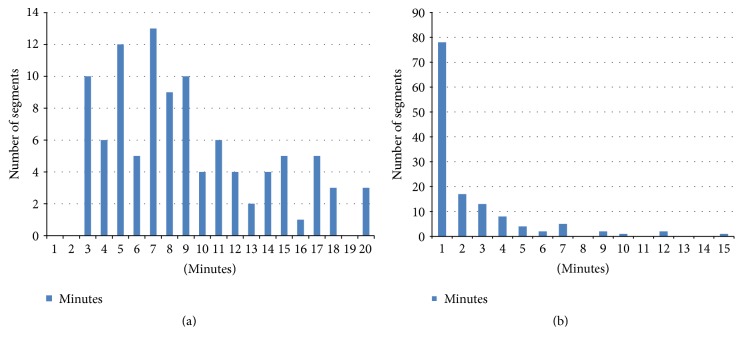
Distribution of highly (a) and low correlated (b) segment lengths for all subjects after synchronization procedure.

After running the synchronization process, we got segments of highly correlated data. Figure 1 shows how the lengths of signal segments are distributed. We can see that most segments are 3–18 minutes long. The longest segment that is highly correlated with the gold standard data is 110 minutes long. The default parameter settings minimize the number of overly short (<5 min) segments. Most of the bad segments (Figure 3) are shorter than one minute, and only one bad segment was 60 minutes long.

The synchronization procedure resulted in highly (>97%) correlated synchronized data segments with various durations.  Table 1 shows the overall duration of signals. Subject #1 had the lowest usable time with only 2 hours and 6 minutes. The most probable reason for such a low time is the chest hair which reduced the contact between electrodes and the skin. For this reason, this subject was excluded from calculation of average results.

Table 2 shows results in time domain for the Schiller and the CardioSport devices after using our algorithm for the synchronization of signals. The time-domain analysis shows pretty close values for both mean RR values and standard deviation. The formula used for computing the standard deviation of RR intervals is as follows:

(2) $$\begin{array}{c} \text{STD }RR=\sqrt{\frac{1}{N-1}\overset{N}{\underset{j=1}{\sum }}{\left({RR}_{j}-\overset{-}{RR}\right)}^{2}}. \end{array}$$

Average mean RR values for Schiller and CardioSport devices are 850.80 and 870.69, respectively. Average STD RR for the Schiller device is 108.42 and it is 110.93 for the CardioSport device.

The frequency-domain analysis is presented in Table 3. The absolute power was compared for very low frequency (VLF: 0–0.04 Hz), low frequency (LF: 0.04–0.5 Hz), and high frequency (HF: 0.15–0.4 Hz) and ratio between low frequency and high frequency (LF/HF). The results show no significant difference between Schiller and CardioSport device values.

The average mean absolute percentage error (MAPE) between the two signals is 2.32% with a high average correlation of 99.67%.

### 3.2. Device Validation Study: Data Cleaning Method

We run the data cleaning algorithm described in Section 2 on the data recorded by the chest belt. The duration of the resulting signal is shown in Table 4. Similar to the synchronization process, we got a very short duration for one subject and we excluded this subject from further analysis. It is important to note that, due to the noise on Schiller device records, we had to remove noisy parts from the “gold standard” signal as well. Therefore, even though the signal was recorded for 12 hours continuously, the overall duration is much less. The calculation shows that, in the worst scenario, only 45% of the signal can be used for analysis using this data cleaning method. However, in the best scenario, this number reaches 95%. This leads to a conclusion that results are quite subject dependent.

Table 5 shows the results of data analysis in the time domain after removing bad parts with the data cleaning algorithm. We can see that the mean RR intervals for the Schiller and the CardioSport devices are 851.14 and 871.23 and the standard deviations are 104.61 and 106.35, respectively. In general, the CardioSport device has slightly greater values but they are very close.

The minimum, maximum, and average percentage errors on the whole signal were calculated using a 5 minutes long sliding window with one-minute shift step (Table 6). Only one subject had a very high maximum error value of 33.86%. By visual examination, we determined that the cause of this high error percentage was in fact the presence of artifacts in the “gold standard” Schiller device measurements. Despite that, average error values are at a very low level of 2.20%.

**Figure 4:**
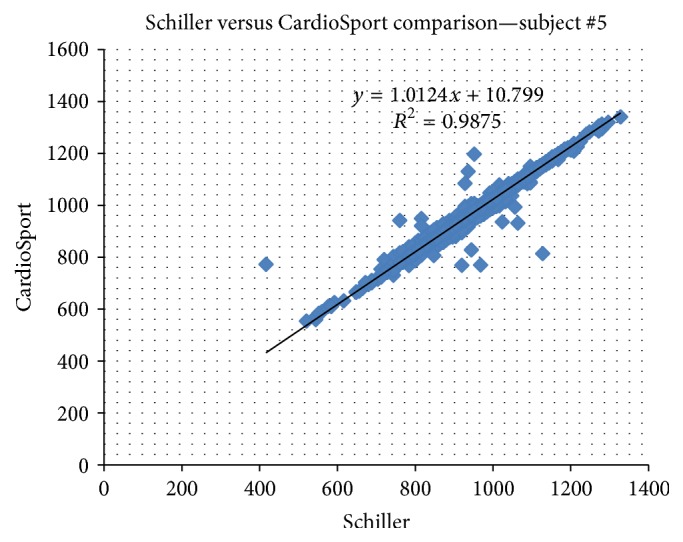
Comparison of CardioSport and Schiller device after data cleaning.

Figure 4 demonstrates the typical relationship between the CardioSport and Schiller measurements using a scatter plot for subject #5. All slope values are close to 1. The lowest slope value is 0.9757, while the highest value is 1.0184. The average mean absolute percentage error (MAPE) between the two signals was 2.62% with a high average correlation of 98.76%.

### 3.3. Clinical Study

Five subjects were excluded from further analysis because they reported a decrease (instead of the expected increase) of their stress level while playing the Stroop game. As an explanation, some participants reported that playing the game was much more joyful than relaxation music. Others reported that the game kept their mind focused and that the relaxation music brought them back to their problems and duties of the day. Some also reported anxiety about the experiment itself which vanished while playing. After removing these records, we run the data cleaning algorithm which identified 10 noisy records, probably due to too much movement. These were also excluded, so the active dataset decreased to 31 subjects' records (20 men and 11 women; average age = 24.7 years).

Table 7 shows *p* values of the Wilcoxon paired-samples test, for the relax versus stress parts, for the relevant HRV features (*n* = 31).

We found a statistically significant difference for the following time-domain features: mean RR (*p* = 0.0001), mean HR (*p* = 0.0001), pNN50 (*p* = 0.0103), NN50 (*p* = 0.0128), RMSSD (*p* = 0.0255), and HRV triangular index (*p* = 0.0456). In frequency domain, two features showed statistically significant difference: HF (ms^2^) with *p* = 0.0054 and LF (ms^2^) with *p* = 0.0128. The VLF (%) feature was also close but not significantly different (*p* = 0.0745). In nonlinear analysis, the SD1 feature showed a statistically significant difference (*p* = 0.0268). The average percentage differences and the minimum percentage differences are shown in Table 8.

Table 9 shows the correlations between the important features during the relaxation part of experiment. We can see very high positive correlation (higher than 0.9) between the following features: NN50 and RMSSD (0.94818), pNN50 and RMSSD (0.935664), and pNN50 and NN50 (0.98966).* Poincaré* plot SD1 feature was highly correlated with HF (ms2) feature. Only one very high negative correlation was found between features mean RR and mean HR (−0.99452).

**Figure 5:**
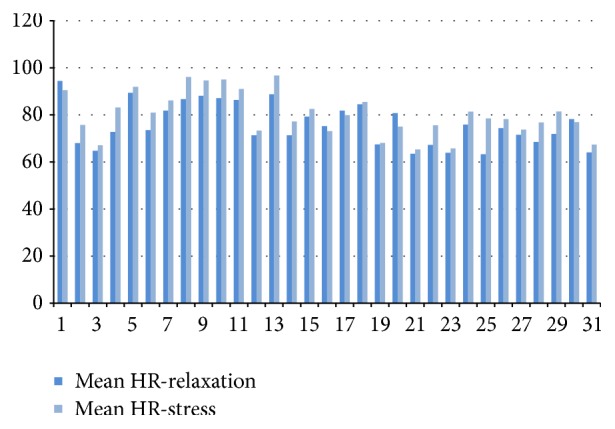
Mean HR feature for all subjects during relaxation and while playing game.

Figure 5 shows, as an example, the values of an observed feature (mean HR) for the relaxation period and the game playing period, respectively.

### 3.4. Clinical Study: Stress Detection Performance Compared to Other Methods

The accuracy, sensitivity, and specificity values for correctly detecting stress are shown in Table 10 for our algorithm, the linear algorithm proposed by Melillo et al., and the nonlinear algorithm proposed by the same authors.

## 4. Discussion

Stress is a very complex subject and measuring stress is not an easy task. There are many markers that could be used, many algorithms that could be applied, and many forms of stress which could be observed. Heart rate variability, being simple and noninvasive, has recently become one of the most popular methods for detecting stress. Still, this is not an easy task, since HRV is not a single value; rather, it consists of many features that can be observed in time domain and frequency domain or using nonlinear analysis. The literature generally reports that, under mental stress, the mean RR, pNN50, STD RR, and RMSSD features decrease, while the mean HR and LF features increase significantly. However, significant differences for the same features and sometimes even opposite results (e.g., LF feature) are also reported. One probable cause for this inconsistency in literature could be the fact that stress is not the only condition that influences changes in HRV. Physical activity, body posture, breathing, age, gender, and illnesses all have a great influence on HRV. In this paper, we analyzed various HRV features in order to find those that change significantly under mental stress and proposed a simple stress detection algorithm.

In the device validation study, we tested the reliability of a low cost heart rate meter. The CardioSport TP3 heart rate meter device was used simultaneously with a professional ECG recorder (Holter) device. We compared the results using standard deviation, correlation, and scatter plot diagram with slope of the regression line which are commonly used in literature [36, 40, 50]. However, before we analyzed the results, we used a simple data cleaning algorithm to eliminate noisy parts without using any data correction. The data cleaning process reduces the overall duration of the signal but it increases its quality. After data cleaning, all results of the CardioSport device were very close to the Schiller device with an average correlation of 98.73%. The downside of the data cleaning algorithm is that it will delete sections of the signal even with the slightest detachment. Hopefully, with advance in wearable sensors, new forms of heart rate monitors like rings or bracelets with firmer attachment to body will be available.

In the next step, we demonstrated how a simple mental stressor can influence HRV features significantly. Our findings are not very different from previous research, showing that HRV can indeed be used as an indicator of mental stress. We found that, under the influence of mental stress, mean HR increased, while mean RR, pNN50, RMSSD, and HRV triangular index decreased. Contrary to the results from literature, we did not find a statistically significant difference in STD RR feature (*p* = 0.1583). This could be explained by the fact that we analyzed only 10 minutes in each part of the experiment, while STD RR feature describes long-term variability. A limitation of our study is that we only analyzed the influence of mental stress. Physical or emotional stress could influence observed features in a completely different way.

For some subjects, the experiment failed to provoke mental stress which is of course a shortcoming of the experiment setup; however, it would be very hard to design a method that is successful in all cases. The Stroop test was chosen because it is easy to implement and is generally accepted in the literature for such purposes. Since it increases the speed of the game proportionally with the user's results, it should increase the stress level regardless of the subjects' cognitive level. We believe that other factors, such as the subject's prior experience and motivation for playing computer games, are harder to control.

As a conclusion of this study we created a robust stress detection algorithm. Unlike other stress detection algorithms which use several stress markers [51, 52], we used only HRV features for stress detection but with relatively high stress identification ratio. We were able to get an accuracy rate of 74.60%, somewhat below the 85% reported by the algorithm in [53]; however, the latter uses the full electrocardiogram (ECG) measurement compared to using only RR intervals in our case.

If we compare our algorithm with other algorithms for stress detection using only HR and HRV parameters, we can say that we achieved higher identification rate than the algorithm in [43] and a worse result than [48] or [45] and around 15% worse compared to [44, 47] but we used only time-domain features for stress identification instead of frequency-domain features or using nonlinear analysis which are much more expensive to calculate.

Since performance of particular algorithm depends on multiple parameters like type of stressor, number of subjects, methods used, and so forth, we compared the performance of our algorithm with two state-of-the-art algorithms. Although our algorithm showed lower declared accuracy, the comparison of its performance on the same dataset showed much better results than the algorithm that uses nonlinear HRV features [44] and slightly better performance than the algorithm that uses linear HRV [45] features for stress detection. We think that the poor performance of the two tested methods could partly be due to the fact that our stressor, the Stroop game, was not as strong as the university exam stressor used for the development of the Melillo algorithms or driving [48]. Such results show that the same algorithm can give very different results on different dataset meaning that comparing strictly by accuracy values is not good indicator if comparison is not performed on the same dataset.

A weakness of the proposed algorithm is that it only detects events that provoke stress for a particular subject. It cannot classify the current state of a subject in given moment. However, this was not the intention of our algorithm from the beginning and we propose using combination of our algorithm with typical classification algorithms for achieving greater insight into stressful events and a subject's current state.

A strength of our algorithm is that an event or a series of stressful events could be detected before entering into a stressful state defined by classification algorithms. The user could be informed by sound, vibration, or other kinds of alert when stressful event happens, leading to greater awareness about daily stressors. Also, this algorithm is very simple and easy to implement in mobile environments due to the fact that all HRV features are chosen only from time domain.

## 5. Conclusions

From this research we can conclude that even a simple low cost heart rate monitor device can detect features that change significantly under the influence of mental stress. Using these results we created a simple stress detection algorithm that is being integrated in the Lavinia lifestyle counseling mobile application [54] for further testing and refinement in real-life stress situations. If stress detection proves to be reliable for larger samples, it will be used in the blood glucose prediction models developed for diabetics.

## Figures and Tables

**Table 1:** Signal durations after the synchronization process.

Subject number	#1	#2	#3	#4	#5

Duration (hh:mm:ss)	2:06:18	10:53:28	8:45:40	10:30:17	7:46:56

**Table 2:** Time-domain results after the synchronization process.

Subject number	Mean RR (ms)			STD RR (ms)		
	Schiller	CardioSport	Error	Schiller	CardioSport	Error
#1	738.27	755.47	2.28%	123.34	125.09	1.40%
#2	704.04	720.42	2.27%	91.35	93.47	2.27%
#3	907.63	928.88	2.29%	90.40	92.83	2.62%
#4	854.53	874.50	2.28%	144.74	148.00	2.20%
#5	937.01	958.97	2.29%	107.18	109.41	2.04%
Average	850.80	870.69	2.28%	108.42	110.93	2.11%

**Table 3:** Frequency-domain analysis after the synchronization process.

Subject number	Schiller				CardioSport				Error			
	Absolute power (ms^2^)				Absolute power (ms^2^)				%	%	%	%
	VLF	LF	HF	LF/HF	VLF	LF	HF	LF/HF	VLF	LF	HF	LF/HF
#1	7937.6	3086	1578	1.956	8444	3224	1330	2.4235	6.00	4.28	18.65	19.29
#2	5431.5	626.6	245	2.557	5723	659.3	250.9	2.6281	5.09	4.96	2.35	2.71
#3	4251.2	1927	494.4	3.898	4543	2055	538.8	3.8146	6.42	6.23	8.24	2.19
#4	12682	1790	636.5	2.813	13514	1869	621.5	3.0077	6.16	4.23	2.41	6.47
#5	6139.8	1212	476.7	2.542	6465	1274	481.4	2.6459	5.03	4.87	0.98	3.93

**Table 4:** Signal durations after the data cleaning process.

Subject number	#1	#2	#3	#4	#5
Duration (hh:mm:ss)	1:28:10	11:20:03	6:15:38	9:27:07	4:29:44

**Table 5:** Time-domain analysis after the data cleaning process.

Subject number	Mean RR (ms)			STD RR (ms)		
	Schiller	CardioSport	Error	Schiller	CardioSport	Error
#1	707.80	724.03	2.24%	136.04	138.63	1.87%
#2	700.40	716.70	2.27%	91.33	93.24	2.05%
#3	899.20	920.97	2.36%	99.67	99.77	0.10%
#4	846.46	866.25	2.28%	139.26	142.33	2.16%
#5	958.49	981.00	2.29%	88.16	90.08	2.13%
Average	851.14	871.23	2.29%	104.61	106.35	1.66%

**Table 6:** Minimum, maximum, and average percentage error.

Subject number	Minimum error	Maximum error	Average error
#1	0.08%	3.50%	1.50%
#2	0.01%	7.71%	2.12%
#3	0.04%	33.86%	3.22%
#4	0.13%	6.72%	1.92%
#5	0.07%	5.11%	2.22%
Average	0.06%	13.35%	2.37%

**Table 7:** Statistical significance of the observed features ordered by *p* value.

Feature	*p* value
*Mean RR*	*0.0001*
*Mean HR*	*0.0001*
*HF (ms* ^*2*^)	*0.0054*
*pNN50*	*0.0103*
*NN50*	*0.0128*
*LF (ms* ^*2*^)	*0.0128*
*RMSSD*	*0.0255*
*Poincaré plot, SD1*	*0.0268*
*HRV triangular index*	*0.0456*
VLF (%)	0.0745
STD RR	0.1583
HF (%)	0.1583
Poincaré plot, SD2	0.2725
LF/HF	0.4565
VLF (ms^2^)	0.4565
TINN	0.5967
Power (n.u.)-HF	0.7390
Power (n.u.)-LF	0.7539
STD HR	0.9687

**Table 8:** Average percentage difference and minimum percentage difference for the features computed from the HR signal.

Feature	Average percentage difference	Minimum percentage difference
Mean HR	6.88	0.94
RMSSD	27.86	3.98
pNN50	72.76	3.88

**Table 9:** Correlation of observed features during relaxation part.

	Mean RR	Mean HR	RMSSD	NN50	pNN50	HRV t.i.	LF (ms^2^)	HF (ms^2^)	P.P., SD1
Mean RR	1.00								
Mean HR	−0.99	1.00							
RMSSD	0.29	−0.28	1.00						
NN50	0.38	−0.37	0.95	1.00					
pNN50	0.48	−0.47	0.94	0.99	1.00				
HRV t.i.	0.25	−0.25	0.78	0.77	0.75	1.00			
LF (ms^2^)	0.08	−0.09	0.15	0.17	0.16	0.28	1.00		
HF (ms^2^)	0.20	−0.20	−0.09	−0.03	0.00	−0.21	0.33	1.00	
P.P., SD1	0.12	−0.13	−0.01	0.01	0.03	−0.15	0.51	0.89	1.00

**Table 10:** Performance comparison of the three stress detection methods.

Feature	Melillo linear	Melillo nonlinear	Our method
Accuracy	61.29%	50.00%	74.60%
Sensitivity	61.29%	29.03%	75.00%
Specificity	61.29%	70.97%	74.19%

## Appendix

### A. The Instructions for the Clinical Study

*Experiment: Measuring Physiological Effects of Heart Rate Parameters during Various Situations*

*Instructions to Participants*. During this experiment we will measure physiological parameters of various situations. First, we will measure parameters during listening to relaxation music and then we will measure the same parameters during playing simple Android based game using mobile phone. You will need to fill data in the questionnaires three times: before experiment starts, after the relaxation music stops, and at the end of experiment. If you feel any discomfort during this experiment, you can stop at any time:

*Personal Data*

Name or code: —
Age: —
Gender: M/F

*How to Play*. In this game screen is divided in four parts. In each part word is shown with different color. Words are color names like red, blue, purple, black, green,…. You need to touch the word which is colored in the color of the word. For example, if word red is colored with black, then you skip it, but if the word red is colored in red color, then you need to press that word. If you make a mistake, game ends. You need to get as high score as possible. For every choice, you have only a limited amount of time. If time runs out, game is over. You can play as many games as you can during 10 minutes:

*Example*

Word red is colored red so this is* correct* choice.
Word green is colored with black color so this is* incorrect* choice.
Word blue is colored with orange color so this is* incorrect* choice.
Word black is colored with blue color so this is* incorrect* choice.

### B. Experiment Data and Questionnaire

*Experiment Data*

Date: —
Protocol
  Give person the paper with experiment description. Let him/her read whole description (including “how to play” section). Ask if they understand the experiment. Tell the person that in this experiment they will first listen one relaxation song and then they will play simple game. Show the person how to play game shortly if necessary. Explain to person that during whole experiment he/she will need to wear chest belt for collecting heart rate data.
  Ask the person to put chest belt. Ensure privacy for person to put chest belt on.
    Time (hh:mm): —
  Establish bluetooth connection between chest belt and Android device. Write down time when this occurs.
    Time (hh:mm): —
  Ask the person about current perceived stress level from 1 to 10 where 1 is no stress and 10 is maximum stress level and write down perceived stress level
    Time (hh:mm): —
    Perceived stress level: —
  Tell the person to relax while listening relaxation music. Start playing relaxation music and write the exact start time (when you hit the play button)
    Time (hh:mm): —
  When music stops, write down the time of the event. Ask the person about perceived stress level after listening the song from 1 to 10 where 1 is no stress and 10 is maximum stress level. Write down perceived stress level
    Time (hh:mm): —
    Perceived stress level: —
  Ask person to play Android based game for the next 10 minutes. Write the start time and set countdown timer to at least 10 minutes.
    Time (hh:mm): —
  At the end of 10 minutes, tell person to stop playing and write down the time. Ask person about perceived stress level after playing game from 1 to 10 where 1 is no stress and 10 is maximum stress level. Write down perceived stress level
    Time (hh:mm): —
    Perceived stress level: —

## Abbreviations

AAL: Ambient assisted living
BP: Blood pressure
ECG: Electrocardiograph
EMG: Electromyogram
GSR: Galvanic skin response
HF: High frequency
HR: Heart rate
HRV: Heart rate variability
LF: Low frequency
MAPE: Average mean absolute percentage
NN50: The number of pairs of successive beat-to-beat or NN intervals that differ by more than 50 ms
pNN50: The proportion of NN50 divided by total number of NNs
RMSSD: The square root of the mean of the squares of the successive differences between adjacent intervals
RR (interval): R wave to R wave interval
VLF: Very low frequency.
